# Fibroadhesive scarring of grafted collagen scaffolds interferes with implant–host neural tissue integration and bridging in experimental spinal cord injury

**DOI:** 10.1093/rb/rbz006

**Published:** 2019-02-04

**Authors:** Haktan Altinova, Sebastian Hammes, Moniek Palm, Jose Gerardo-Nava, Pascal Achenbach, Ronald Deumens, Emmanuel Hermans, Tobias Führmann, Arne Boecker, Sabien Geraldine Antonia van Neerven, Ahmet Bozkurt, Joachim Weis, Gary Anthony Brook

**Affiliations:** 1 Department of Neurosurgery, RWTH Aachen University Hospital, Aachen, Germany; 2 Institute of Neuropathology, RWTH Aachen University Hospital, Aachen, Germany; 3 Police Headquarters Berlin, Medical Commission, Berlin, Germany; 4 Institute of Neuroscience, Université Catholique de Louvain, Brussels, Belgium; 5 Donnelly Centre for Cellular & Biomolecular Research, University of Toronto, Toronto, Canada; 6 Department of Hand-, Plastic and Reconstructive Surgery, Burn Centre Trauma Centre, BG Trauma Centre Ludwigshafen, University of Heidelberg, Ludwigshafen, Germany; 7 Department of Plastic, Reconstructive and Hand Surgery, Burn Centre, RWTH Aachen University Hospital, Aachen, Germany; 8 Department of Plastic, Aesthetic, Hand and Burn Surgery, Helios University Hospital Wuppertal, University Witten/Herdecke, Wuppertal, Germany

**Keywords:** spinal cord injury, CNS-scarring, type-I collagen, scaffold, astrogliosis, fibrosis, encapsulation

## Abstract

Severe traumatic spinal cord injury (SCI) results in a devastating and permanent loss of function, and is currently an incurable condition. It is generally accepted that future intervention strategies will require combinational approaches, including bioengineered scaffolds, to support axon growth across tissue scarring and cystic cavitation. Previously, we demonstrated that implantation of a microporous type-I collagen scaffold into an experimental model of SCI was capable of supporting functional recovery in the absence of extensive implant–host neural tissue integration. Here, we demonstrate the reactive host cellular responses that may be detrimental to neural tissue integration after implantation of collagen scaffolds into unilateral resection injuries of the adult rat spinal cord. Immunohistochemistry demonstrated scattered fibroblast-like cell infiltration throughout the scaffolds as well as the presence of variable layers of densely packed cells, the fine processes of which extended along the graft–host interface. Few reactive astroglial or regenerating axonal profiles could be seen traversing this layer. Such encapsulation-type behaviour around bioengineered scaffolds impedes the integration of host neural tissues and reduces the intended bridging role of the implant. Characterization of the cellular and molecular mechanisms underpinning this behaviour will be pivotal in the future design of collagen-based bridging scaffolds intended for regenerative medicine.

## Introduction

Severe traumatic spinal cord injury (SCI) causes a devastatingly rapid and permanent loss of function, largely due to destruction of spinal tissue, including long-distance projecting axons in white matter tracts, resulting in the effective separation of projection neurons from their target neurons [1]. Experimental and post-mortem studies have shown that such injury-induced primary and secondary degenerative events include apoptotic cell death, inflammation and oedema, resulting in the formation of cystic cavitation as well as glial and connective tissue scarring [2–6]. These events lead to the development of molecular and physical barriers that are hostile to axon regeneration and prevent any significant functional recovery [7–10].

Numerous experimental strategies have shown promise in promoting tissue repair and functional recovery, including blockade of myelin-associated axon growth-inhibitory molecules, enzymatic degradation of axon-repulsive extracellular matrix-related molecules (e.g. highly sulphated chondroitin sulphate proteoglycans (CSPGs)), and implantation of axon growth-promoting glia, stem cells or their derivatives (for recent reviews; see [11–13]). It is anticipated that bioengineered scaffolds are likely to contribute to future, multi-component repair strategies by the importation of longitudinally orientated materials that can substitute for the damaged portions of white matter tracts and provide a bridge across scar- and cavity-rich lesion sites [14].

Collagen-based scaffolds have proven to be particularly attractive in tissue engineering and regenerative medicine because of the natural abundance of collagen, its ability to be engineered into almost any 3D form, and its inherent biological functionality and biodegradability (depending on the extent of cross-linking) [15, 16]. Such collagen-based scaffolds have shown great promise as cell carriers and bridging devices in numerous experimental models of both central nervous system (CNS) and peripheral nervous system (PNS) injury [17–24]. Our earlier *in vitro* investigations have demonstrated the biocompatibility of a longitudinally microporous type-I collagen scaffold with a range of neuronal, glial and fibroblastic cell types [25–28]. Implantation of the scaffold into critical sized defects of the rat PNS have supported significant repair in experimental animal models [23, 29–31] and have been demonstrated to be tolerated and safe when implanted in clinical trials [32]. Implantation of similar type-I collagen scaffolds into experimental unilateral resection injuries of the adult rat cervical spinal cord have induced a statistically significant improvement of forelimb motor function in the absence of substantial axonal growth or astroglial integration into the scaffold [31]. Attempts to improve implant–host integration by seeding the scaffold with axon growth-promoting olfactory nerve ensheathing cells prior to implantation induced a tendency for improved axonal growth, but this failed to reach the level of statistical significance and no further enhancement of function could be seen [33]. The implanted collagen scaffold was unable to support a clear and consistent degree of host neural tissue integration that would be required to bridge the lesion site efficiently. In this context, the host scarring response appears to have played a major role, despite the fact that quantitative morphometric analyses indicated a reduction of reactive astrogliosis around the implant [31]. To date, most investigations on scaffold-host integration following implantation into the lesioned spinal cord have predominantly focussed on the role of the astroglial component of the scarring response (e.g. [34]). The roles played by cells involved in the (equally important) fibroadhesive component of scarring seem to have been largely over-looked.

In the present study, we demonstrate the involvement of a non-astroglial (i.e. fibrotic) component to the scarring response that takes place at the implant–host interface following implantation of a highly microstructured type-I collagen scaffold into an experimental model of acute SCI. Furthermore, we demonstrate how the presence of such fibroadhesive scarring can have a significant impact on quantitative estimates of the extent of astrogliosis in the surrounding reactive host tissues. A clear understanding of both astroglial and fibroadhesive aspects of the scarring response at the implant–host interface will facilitate the future development of such collagen-based scaffolds for use in tissue engineering and regenerative medicine following traumatic injury to the spinal cord.

## Materials and methods

### Experimental animals

All animal experiments were carried out at the Institute of Neuroscience, group of Neuropharmacology, Université Catholique de Louvain (UCL), Belgium, according to the EU directive of 22 September 2010 (2010/63/EU) and with the approval of the local ethical committee on animal experimentation (2014/UCL/MD/012) as well as that of the Belgian authority on animal experimentation (LA 1230618).

The experiments were performed on adult female Sprague-Dawley rats (*n* = 24, body weight 180–200 g), obtained from the local breeding facility at the UCL. Animals were housed in standard makrolon cages (in groups of two to three per cage) and were allowed free access to food and water. Lighting conditions followed a 12:12 h light–dark cycle. In all cases, every attempt was made to minimize the number of animals used in the investigations as well as any pain or discomfort.

### Surgical procedure

The unilateral resection injury of the lateral cervical spinal cord white matter (with or without the implantation of the type-I collagen scaffold) has been described previously Briefly, a sub-cutaneous injection with buprenorphine (Temgesic 0.1 mg/kg body weight) was given to all animals 30–60 min prior to surgery. Anaesthesia was induced by inhalation of a 4–5% mixture of isoflurane in air and maintained at 2% using a U-400 anaesthesia unit (Agntho’s, Lidingö, Sweden). Ophthalmic ointment was applied to prevent corneal drying. The neck and shoulder area was shaved and disinfected before making a mid-line incision of the skin. After blunt dissection of the dorsal neck musculature, a hemi-laminectomy of C3–C4 vertebrae was performed to expose the spinal cord. A small dural window was opened, followed by a 2-mm long right-sided lateral funiculotomy using micro-scissors. Completeness of the funiculotomy was verified microscopically following aspiration of the lesion site. Care was taken to protect major dorsal spinal cord blood vessels. The lesion site was then either left untreated or filled with an appropriately shaped longitudinally micro-structured type-I collagen scaffold (Matricel GmbH, Herzogenrath, Germany). The dural flap was repositioned using 10/0 sutures (Ethicon, Inc., Somerville, USA), the overlying muscles were closed in layers and the skin sutured (4/0 Prolene^®^, Ethicon, Inc.). Experimental animals were divided into two groups: (i) those only receiving a lesion (control group, *n* = 12 animals) and (ii) those receiving a lesion followed by implantation of the longitudinally micro-structured type-I collagen scaffold (experimental group, *n* = 12 animals). The spinal cords of eight animals per group were used for morphometric analyses of longitudinal cryosections, while the spinal cords of the remaining four animals were used for immunohistochemistry of transverse cryosections.

### Spinal cord tissue processing and histological analyses

After a survival time of 10 weeks post-surgery (wps), animals were transferred into a euthanasia chamber that was flushed with carbon dioxide until respiratory arrest. Animals were fixed by transcardial perfusion with 100 ml phosphate buffered saline (PBS, pH 7.4) followed by ∼300 ml of cold 4% paraformaldehyde (PFA in 0.1 M phosphate buffer). Spinal cords were carefully dissected and post-fixed in the same fixative for 24 h. They were then cryoprotected (30% sucrose in PBS at 4°C) and 1 cm long tissue blocks, centred around the lesion/implantation site, were excised, frozen in dry-ice and cut in longitudinal or transverse section (25 and 10 µm thick, respectively) using a cryostat (Leica CM3040S). Serial sections were mounted onto adjacent SuperFrost-Plus slides (R. Langenbrinck GmbH, Germany), such that sampling distances of 250 μm were obtained between adjacent longitudinal sections on the same slide and 200 μm distance between adjacent transverse sections. The slides were then stored at −80°C prior to histological or immunohistochemical processing. Longitudinal sections were stained with haematoxylin and eosin (H&E) for general histological observations.

Prior to immunohistochemistry, antigen retrieval was performed by heating sections in citrate buffer (pH 6) for 5 h at 37°C. All solutions were prepared in antibody diluent consisting of PBS containing 1% bovine serum albumin (Carl Roth GmbH, Germany) and 1% Triton X-100 (Sigma-Aldrich, USA). After washing steps in PBS and a 1 h serum-block incubation in normal goat serum (5%, Jackson ImmunoResearch, UK), sections were incubated overnight at room temperature with the following primary antibodies: rabbit anti-glial fibrillary acidic protein (GFAP, 1:2000, DAKO), rabbit anti-ionized calcium-binding adapter molecule I (IBA-I, 1:1000, Fujifilm Wako Chemicals Europe), rabbit anti-S100 (1:20 000, DAKO), mouse anti-GFAP (Clone GA5, 1:2000, MERCK), mouse anti-200 kDa neurofilament (NF200, clone NE14, 1:2000, Sigma-Aldrich MERCK) and mouse anti-vimentin (clone V9, 1:200, Sigma-Aldrich MERCK). The following day, sections were washed in PBS and incubated with fluorochrome-conjugated secondary antibodies: Alexa-488 conjugated goat anti-rabbit IgG and Alexa-594 conjugated goat anti-mouse IgG (both at 1:500, ThermoFisher Scientific) for 2 h. The DNA intercalator 4′,6-diamino-2-phenylindole (DAPI 1:1000, Sigma-Aldrich) was used to provide a nuclear counterstain. The slides were then coverslipped using DAKO-Mounting Medium (DAKO, Germany). Sections were observed with a Zeiss Axioplan epi-fluorescence microscope connected to a Zeiss AxioVision CCD camera. Images were processed and stored using the Zeiss AxioVision 4.8 software.

### Morphometric analysis of reactive astrogliosis and microglial responses

**Figure 1:**
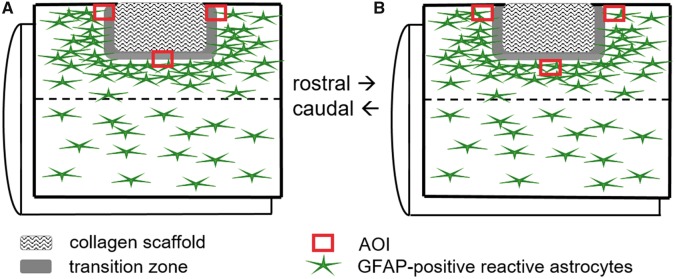
Schematic diagram illustrating the positions of the AOI for morphometric analyses. A lateral funiculotomy of the right cervical spinal cord followed by implantation of the microstructured type-I collagen scaffold or no implantation (lesion only, not illustrated). (**A**) Area of interest (AOI) for the quantification of GFAP-immunoreactive astrogliosis defined by the location at the edges of the implanted scaffold. (**B**) AOI for the quantification of GFAP-immunoreactive astrogliosis defined by the location of the edge of the gliotic tissue

As described previously, two 250 µm spaced sections per spinal cord were examined for morphometric analyses of GFAP-immunoreactivity and IBA-I-immunoreactivity at the lesion– and implantation–host interfaces (control, lesion-only group *n* = 8 animals, scaffold-implanted group *n* = 8 animals) [31, 33]. Figure 1A illustrates the multiple 450× 350 µm areas of interest (AOI) captured for each field (i.e. from rostral-, medial- and caudal interfaces) for which uniform exposure times were used. Schematics in Fig. 1A and B also define the general orientation of the representative illustrated figures. ImageJ^®^ was used to determine the percentage area that was occupied by GFAP-immunoreactive astroglia (as an indicator of the extent of reactive astrogliosis) and by IBA-I-immunoreactive microglia (as an indicator of the extent of the innate inflammatory response). For IBA-I-immunoreactivity, three AOIs (i.e. from rostral, central and caudal) within the collagen scaffold were examined and compared with the corresponding regions of the contralateral (unoperated) side of the spinal cord. For quantitative analyses, a threshold filter was applied that maintained visualization of fine cell processes and branches and the percentage of the AOI that was occupied by immunoreactive profiles was calculated. The results were collated into a Microsoft Excel file and processed for later statistical analysis.

The extent of reactive astrogliosis and activated microglial/macrophage infiltration were compared between groups using an unpaired Student’s *t*-test. The extent of microglial activation and migration into the implanted collagen scaffold was compared with basal levels of IBA-I-immunoreactivity in the respective anatomical position of the contralateral (unoperated) side of the spinal cord using a paired Student’s *t*-test. All graphical data represent the mean ±standard error of the mean (SEM) and was generated using Graph Pad Prism, version 4 (San Diego, CA, USA). A *P* values of 0.05 or less was considered as statistically significant.

## Results

**Figure 2:**
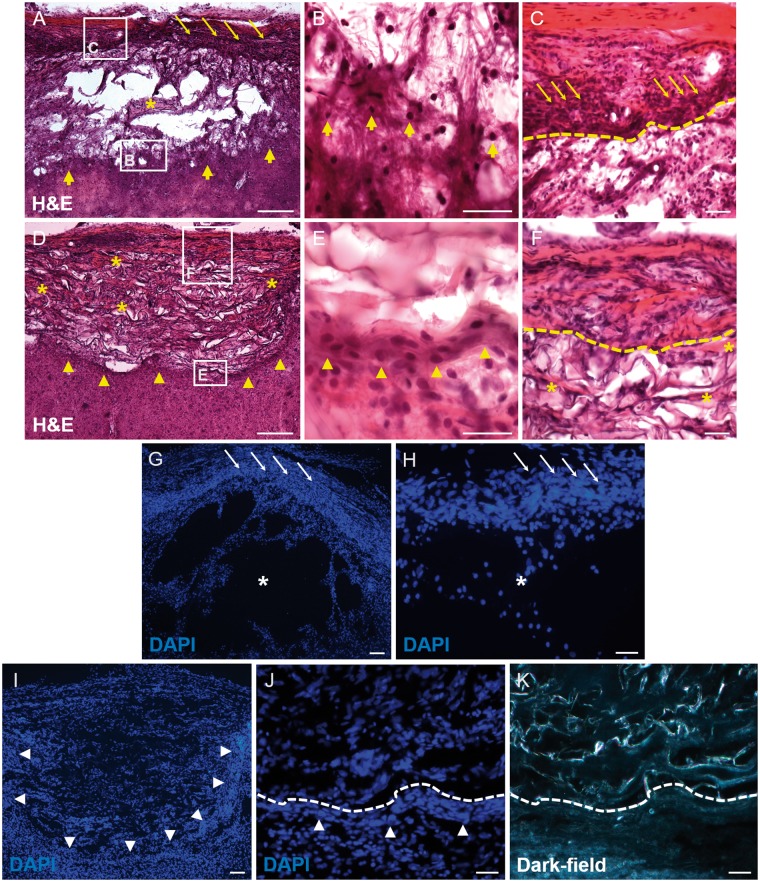
General morphology of the lesion site and implanted collagen scaffolds as revealed by H&E (**A**–**F**) and DAPI staining (**G**–**K**) of longitudinal cryosections. (A) Low-magnification image of large fluid-filled cystic cavities separated by cellular trabeculae (e.g. asterisk) were commonly found in the lesion-only group. At the lateral aspect of the lesion, a band of densely packed cells formed along the medial edge of the dura matter (thin arrows), whereas at the medial aspect of the lesion (thick arrows), no such band of cells could found. Boxed areas in a are shown at higher magnification in (B) and (C). (B) The medial interface of the lesion showing the scattered, rounded haematoxylin-stained nuclei, presumably belonging to reactive astrocytes (thick arrows). (C) Densely packed cells of the lateral tissue bridge (thin arrows), located along the medial edge of the dura mater (intensely red-stained region). the edge of the tissue bridge is indicated by the dashed line. (D) Low magnification of the implanted collagen scaffold that resulted in fewer fluid-filled cystic cavities. In contrast to the lesion-only group, the medial scaffold interface showed a narrow band of clustered cells (arrowheads). Boxed areas in D shown at higher power in (E) and (F). (E) The area immediately adjacent to the implanted collagen scaffold revealed a narrow region of densely packed cells with ovoid nuclei, the orientation of which tended to follow the line of the scaffold–tissue interface (arrowheads). (F) Lateral tissue bridge located along the medial edge of the dura mater (lost during processing) and the outermost edge of the implanted collagen scaffold (dashed lined). (G) Low-magnification image of DAPI-stained lesion site revealing the large cystic cavities (asterisk) and the laterally positioned cellular bridge (thin arrows), seen at higher magnification in (H). (I) Low-power image showing the densely packed cell nuclei at the implanted scaffold–tissue interface (arrowheads). (J) Higher magnification of the band of densely packed cells (arrowheads) in the transition zone following the edge of the implanted collagen scaffold (dashed line). (K) Dark-field image region shown in (J), used to unambiguously identify the scaffold–implant interface (dashed line). Scale bars (A), (D), (G) and (I): 100 μm; (B), (C), (E), (F), (H), (J) and (K): 50 μm

H&E and DAPI stains of longitudinal sections demonstrated the general histology of the control (i.e. lesion only) and collagen-scaffold implanted areas (Fig. 2A–J). Lesions clearly showed trabeculae of scarring tissue separating multiple cystic cavities by 10 wps (asterisks, Fig. 2A, G and H). A band of densely packed cells could be seen at the lateral most edge of the lesion site, situated along the medial surface of the dura mater (thin arrows in Fig. 2A and G). The rostral-, medial- and caudal interface of the cystic cavity-filled lesion sites were surrounded by cells with rounded basophilic nuclei, presumably of reactive astrocytes (thick arrows in Fig. 2A and B). The implanted collagen scaffold demonstrated the presence of fewer, smaller-sized cystic cavities than had been observed in the lesion-only samples (compare Fig. 2D with Fig. 2A). Furthermore, a conspicuous band of cells could be observed at the rostral–, medial– and caudal implant–host interfaces (arrowheads in Fig. 2D and I also shown at higher magnification in Fig. 2E and J). This band of cells formed a transition zone between the edge of the implanted scaffold (indicated by the dotted line in Fig. 2J and K) and the surrounding tissues. The transition zone was generally composed of tightly packed cells with oval-shaped nuclei (Fig. 2E and J) and was associated with a more intense eosin staining in the H&E-stained sections (Fig. 2E). Cells that migrated into the implant demonstrated elliptical nuclei following the orientation of the scaffolds’ microporous framework (Fig. 2I). In the H&E-stained sections, these cells (probably fibroblasts) were often scattered amongst the red-tinted eosinophilic (collagenous) connective tissue that was deposited within the scaffolds’ framework (asterisks in Fig. 2D) and was particularly noticeable near the lateral edge of the implant (Fig. 2F).

**Figure 3:**
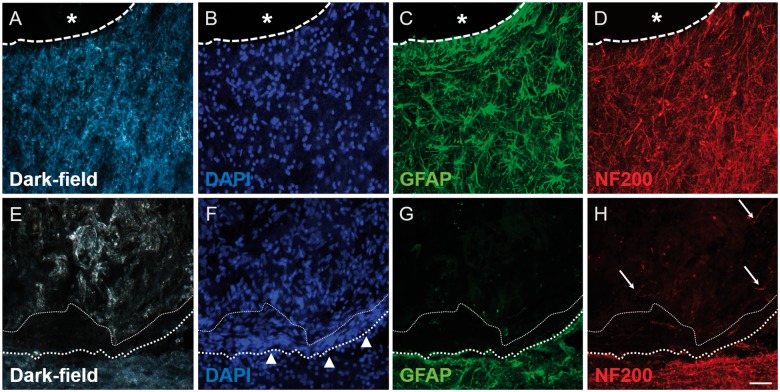
Immunohistochemistry for GFAP and NF200 revealing the tissue interface in lesion-only (A–D) and scaffold-implanted (E–H) animals. (A) Dark-field image and (B) DAPI-stain showing the interface (dashed line) between a fluid-filled cystic cavity (asterisk) and the adjacent intact tissue. (C) GFAP-immunohistochemistry showing the distribution of reactive astrocytes and their processes adjacent to the cystic cavity (asterisk). (D) NF200-immunohistochemistry showing axons located in the same regions as the reactive astrocytes. (E) Dark-field image and (F) DAPI-stain showing the densely cellular band (arrowheads) of the transition zone that forms at the interface between the edge of the implanted scaffold (fine-dotted line) and the adjacent host spinal cord tissue (heavier-dotted line). (G) GFAP-staining reveals that the transition zone is not composed of reactive astrocytes or their processes. (H) Only occasional NF200-positive axonal profiles can be identified within the implanted scaffold (arrows). Scale bar (A)–(H): 50 μm

Immunohistochemistry was used to better define the cellular events taking place in and around the lesion/implantation sites. Dark-field images and DAPI nuclear counterstains were particularly useful in demonstrating the edge of the lesion or the implanted collagen scaffold (Fig. 3A, B, E and F). Strongly GFAP-positive astrocytic cell bodies and processes could be observed extending up to the edge of the injury site in the lesion-only group (Fig. 3C). This distribution was very similar to that demonstrated by NF200-positive axons (Fig. 3D). However, the distribution of GFAP- and NF200-immunoreactivity at the implant–host interface of the scaffold-implanted group was strikingly different (Fig. 3G and H). Astrocytic and axonal profiles only extended up to the outer edge of the dense cellular layer of the transition zone that surrounded the implant (indicated by the bold-dotted line in Fig. 3E–H, the finer-dotted line represents the interface between the cells of the transitional zone and the implanted collagen scaffold). The transition zone was often conspicuous by the absence of GFAP- and NF200-immunoreactivity (Fig. 3G and H) and therefore gave the impression of acting as a barrier to host astroglial and axonal profiles. Only occasional GFAP-positive or NF200-positive profiles could be seen at a short distance within the scaffold or associated with the trabeculae in the cystic cavity filled lesion site of the control group (e.g. arrows for fine, weakly labelled axons in Fig. 3H).

**Figure 4:**
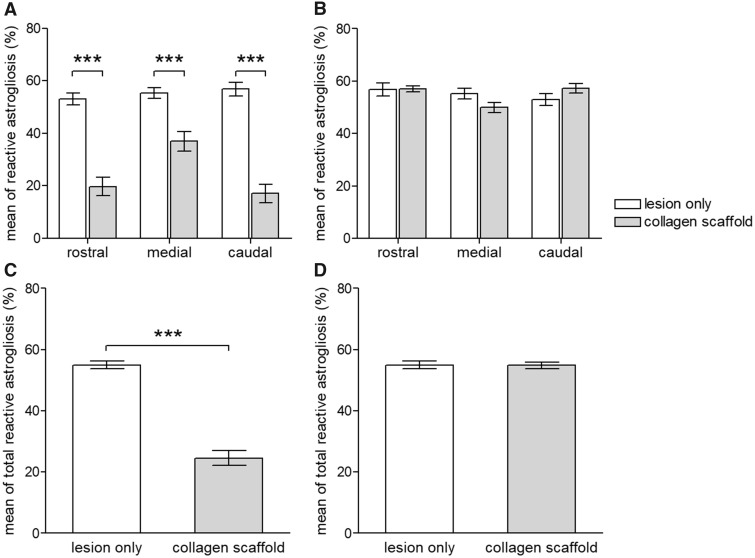
Morphometric analysis showing the clear impact of the choice of positioning of the AOI on the quantification of astrogliosis. (**A**) The positioning of the AOI to coincide with the edge of the implanted scaffold (and thus including the GFAP-negative area of the cellular band around the scaffold) results in an apparent statistically significant reduction of astrogliosis at the rostral-, medial- and caudal interfaces (*P* < 0.001 for each interface). (**C**) The apparent reduction of astrogliosis following the implantation of the collagen scaffold is supported by the summation of data at all implant–host spinal cord tissue interfaces (*P* < 0.001). (**B**) The positioning of the AOI to coincide with the edge of the astroglial scarring (and thus excluding the transition zone around the scaffold) results in no statistically significant differences between the extent of astrogliosis in the lesion-only group and the scaffold implanted group (*P* > 0.05 for the rostral-, caudal- and medial interfaces). (**D**) This interpretation is supported by the summation of the data obtained from all interfaces (*P* > 0.05). values are given as mean±SEM (*P < 0.05; ***P < 0.001)

The extent of reactive astrogliosis surrounding the lesion-only and scaffold-implantation groups was quantified by two slightly different approaches within the same sections. In the first method, the edge of the AOI was chosen to coincide with the edge of the lesion (or the edge of the implanted collagen scaffold as indicated by dark-field microscopy; according to the scheme in Fig. 1A). This sampling method included the densely cellular region of the transitional zone around the scaffold. The resulting morphometric analysis indicated that implantation of the collagen scaffold induced a statistically significant reduction of reactive astrogliosis when compared with the control, lesion-only group (at all rostral, medial and caudal AOI *P *<* *0.001; Fig. 4A; and also for the combined data *P *<* *0.001; Fig. 4C). However, quantification that was based on the positioning of the AOI to coincide with the beginning of the area of reactive astrogliosis (i.e. a sampling method that excluded the densely cellular region of the transition zone; according to the scheme in Fig. 1B) revealed no statistically significant differences in the extent of astrogliosis around the collagen scaffolds in comparison to the control, lesion-only group (*P* > 0.05, Fig. 4B and D).

**Figure 5:**
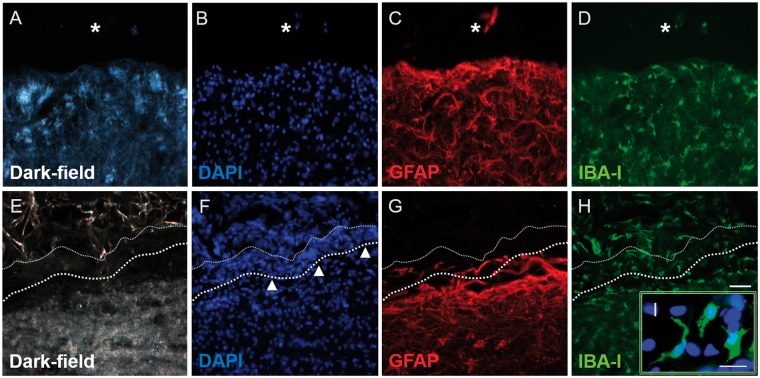
Immunohistochemistry for GFAP and IBA-I revealing the tissue interface in lesion-only (**A**–**D**) and scaffold-implanted (**E**–**I**) animals. (A) Dark-field image and (B) DAPI-stain showing the interface between a fluid-filled cystic cavity (asterisk) and the adjacent intact tissue. (C) GFAP-immunohistochemistry showing the distribution of reactive astrocytes and their processes adjacent to the cystic cavity with occasional processes penetrating the lesion site. (D) IBA-I-immunohistochemistry showing that microglia around the lesion site had largely returned to a ‘resting’, process-bearing morphology. (E) Dark-field image and (F) DAPI-stain showing the dense cellular band (arrowheads) that forms at the interface between the edge of the implanted scaffold (fine-dotted line) and the adjacent host spinal cord tissue (heavier-dotted line). (G) GFAP staining reveals occasional astrocytic profiles penetrating the cellular band around the implanted scaffold. (H) IBA-Iimmunohistochemistry shows that numerous microglia/macrophages have become incorporated into the dense cellular band around the implant. Many of the IBA-I-positive cells within the scaffold (as well as those around the scaffold) have adopted a process-bearing morphology. An example of the (thick) process bearing cells located within the framework of the collagen scaffold is provided in the insert (I). scale bars (A)–(H): 50 μm; (I): 20 μm

IBA-I-immunoreactivity was used to identify the extent of microglial/macrophage involvement in the cellular responses to injury/scaffold implantation. In contrast to the relative paucity of GFAP-immunoreactive astroglial profiles penetrating the collagen scaffold or penetrating the dense band of cells of the transition zone around the implant, substantial IBA-I-immunoreactivity was found within and around the implant (compare Fig. 5C, D, G and H).

**Figure 6:**
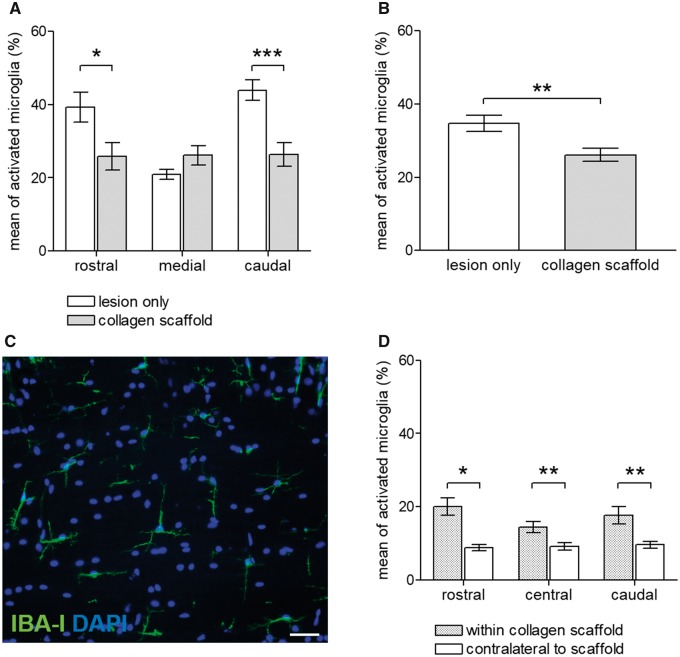
Morphometric analysis of the host microglial/macrophage response to injury and to implantation of the collagen scaffold. (**A**) Quantification of IBA-I-immunoreactivity demonstrated that the greatest degree of residual microglial staining was at the rostral and caudal lesion–host tissue interfaces. The implantation of collagen scaffold significantly reduced the extent of IBA-I microglial/macrophage staining at both rostral and caudal implant–host tissue interfaces (*P* < 0.05 and *P* < 0.001, respectively). (**B**) Summation of the morphometric analyses at the rostral-, caudal- and medial interfaces demonstrated a significant reduction of the host inflammatory response in response to implantation of the collagen scaffold (*P* < 0.01). (**C**) Baseline stain for IBA-I-immunohistochemistry in the contralateral unlesioned spinal cord white matter. Note the small microglial cell bodies with fine, branching processes. (**D**) Comparisons of the IBA-I-immunoreactivity within the scaffold itself with similarly positioned AOI in the contralateral, unlesioned white matter revealed a statistically significant increase of microglia/macrophage within the scaffold. Values are given as mean±SEM (*P < 0.05; **P < 0.01; ***P < 0.001). Scale bar in (C): 50 μm

The majority of the IBA-I-positive profiles within the scaffold (Fig. 5H) were associated with small cell bodies (14–20 µm in diameter) that possessed short and sometimes branched processes (insert Fig. 5I), somewhat reminiscent of the morphology of resting microglia in non-lesioned CNS tissue. Occasionally, multi-nucleated IBA-I-positive giant cells could also be observed within and around the implanted scaffold, but this was a rare phenomenon (not shown). The extent of IBA-I-positive activated microglia at the medial interface of the lesion-only group and scaffold-implanted group was similar (*P* > 0.05, e.g. Figs 5D, H and 6A). However, the rostral- and caudal interfaces of the lesion-only control group demonstrated significantly greater IBA-I-immunoreactivity than could be observed at the rostral and caudal edges of the collagen scaffold-implanted group (*P* < 0.05 and *P* < 0.001, respectively, Fig. 6A). Overall comparisons (including rostral-, medial- and caudal interfaces) indicated a small, but significantly greater IBA-I-immunoreactivity around the lesion-only group in comparison to that of the scaffold implantation group (*P* < 0.01, Fig. 6B). The presence of large, cell-free cystic cavitations within the lesion site of the control, lesion-only group made it impossible to perform any meaningful comparison of the extent of IBA-I-immunoreactivity within the lesion site and within the implanted scaffold. Therefore, IBA-I-positive profiles within the contralateral unlesioned tissues were used as an internal reference (or control) for comparison with the microglial/macrophage distribution within the implanted scaffold. Figure 6C demonstrates the distribution and fine processes of IBA-I-positive microglia in the longitudinal white matter in the normal, resting (baseline) state. The rostral, central and caudal AOI within the scaffold all demonstrated significantly greater IBA-I-positive staining than in the equivalent areas of the contralateral white matter tracts (*P* < 0.05, *P* < 0.01 and *P* < 0.01, respectively, Fig. 6D).

**Figure 7:**
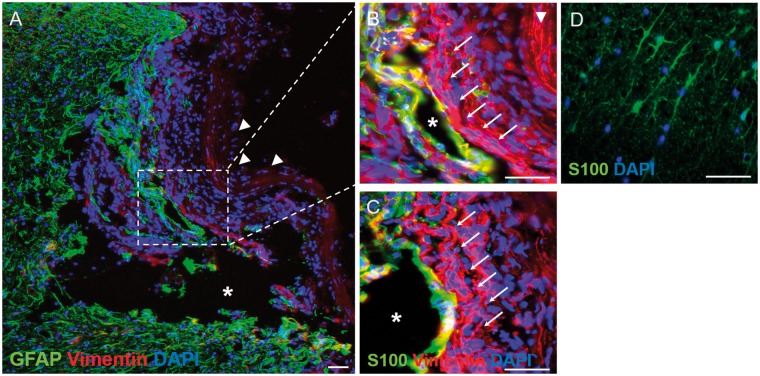
Immunohistochemical demonstration of the distribution of vimentin, GFAP and S100 within the injury site of the lesion-only group. (**A**) Low-magnification overview of the injury site stained for vimentin and GFAP. A large cystic cavity (asterisk) can be seen as well as numerous vimentin-positive/GFAP-negative cells. The lesion site appears to have collapsed slightly but the dura mater can still be seen (arrowheads). A protuberance, containing GFAPpositive cells, can be seen penetrating the lesion site, part of which is shown at higher magnification in (**B**). (B) The GFAP-positive protuberance can be seen surrounding a small cystic cavity (asterisk) and is surrounded by numerous vimentin-positive/GFAP-negative cells that form part of the lateral tissue bridge (arrows), lying next to the inner-most edge of the dura mater (arrowhead). (**C**) A near adjacent section stained for S100 shows that at this particular level, the vimentin-positive/GFAP-positive cells lining the small cystic cavity were also S100-positive suggesting an astrocytic phenotype, however, the vimentinpositive/GFAP-negative cells of the lateral tissue bridge (arrows) are also S100-negative, suggesting a fibroblast-like phenotype. For a comparison with the baseline staining of contralateral, unlesioned white matter astrocytes, see (D). (**D**) Baseline stain for astrocytic S100 in a transverse cryosection in the contralateral unlesioned spinal cord white matter. Note the baseline branching morphology of the S100-positive astrocytic profiles. Scale bars (A)–(D): 50 μm

**Figure 8:**
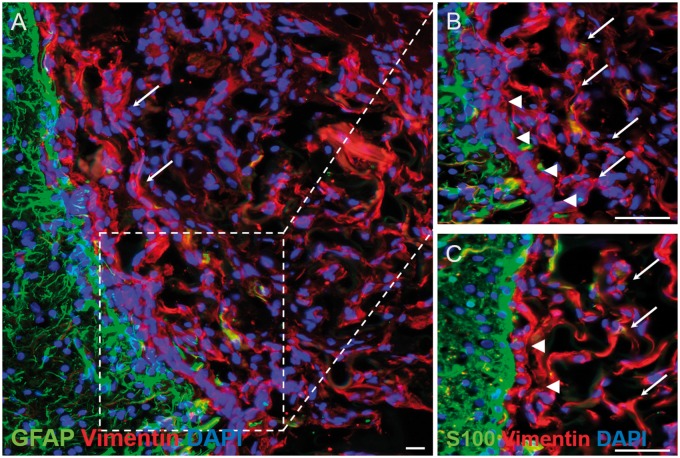
Immunohistochemical demonstration of the distribution of vimentin, GFAP and S100 within the injury site of the collagen scaffold-implanted group. (**A**) Mid-power view of the medial implant–host tissue interface immunohistochemically stained for vimentin and GFAP. The microporous framework of the collagen scaffold is closely associated with numerous, scattered spindle-shaped vimentin-positive/GFAP-negative cells (arrows) that are seen at higher magnification in (**B**). (B) The scattered, spindle-shaped, vimentin-positive/GFAP-negative cells within the scaffold have a similar phenotype to the more densely packed layer of cells (arrowheads) lying in between the scaffold edge and the surrounding host GFAP-positive reactive astrocytes. (**C**) Staining of near adjacent sections for vimentin and S100 demonstrated that the densely packed layer of cells lying between the collagen scaffold and reactive host astrocytes (arrowheads) demonstrated that the cells were S100-negative, suggesting a fibroblast-like phenotype of cells encapsulating as well as penetrating the collagen scaffold. Scale bars (A): 20 μm; (B) and (C): 50 μm

Combinations of vimentin, GFAP and S100 immunofluorescence were used to better characterize the cells within and around the lesion/implant sites. In the lateral connective tissue bridge of the lesion-only group, the vimentin-positive spindle-shaped bipolar cells were largely negative for GFAP (Fig. 7A, arrows in higher magnification image of Fig. 7B) and for S100 (arrows, Fig. 7C), a phenotype that is typical for fibroblast-like cells. This pattern of vimentin-positive, GFAP-negative and S100-negative staining was also demonstrated by the majority of the thin, spindle-shaped cells that migrated into the porous, microchannel framework of the collagen scaffold (arrows, Fig. 8A–C) as well as by the thin bands of spindle-shaped cells of the transition zone, lining the rostral-, medial- and caudal interfaces between the implant and the surrounding host tissue (arrowheads, Fig. 8B and C).

## Discussion

Bioengineered scaffold-based intervention strategies that are intended to bridge traumatic lesions of the spinal cord aim to replace lost or damaged tissue with an orientated, axon growth-promoting substrate. The efficiency of such scaffolds to bridge injury sites critically depends on their ability to integrate with damaged host tissues [18, 19]. Amongst the wide range of natural polymers used for tissue engineering and regenerative medicine in nervous tissue repair (of both CNS and PNS), collagen has proven to be a popular choice ([35–40] also reviewed in [15]). A number of research groups have implanted micro-structured collagen scaffolds into complete transection or hemisection injuries of the adult rats spinal cord (with or without added pharmacological agents, neutralizing agents or seeding with axon growth-promoting cells) but have largely failed to demonstrate any substantial penetration of the scaffold by host astroglia or crossing of the scaffold by regenerating intrinsic CNS axons [18, 19, 31, 33, 37, 40].

The present study has focussed on characterizing the host cellular response to the implanted type-I collagen scaffold and has revealed subtle, easily over-looked differences between the fibroadhesive response within the scaffold and that taking place at the implant–host interface: fibroblasts appeared to be generally loosely scattered throughout the implant whereas an often dense, multi-cellular layer of fibroblast-like cells formed at the transition zone at the implant–host interface. These observations support and extend earlier reports of Cholas *et al.* who implanted longitudinally orientated type-I/III collagen scaffolds wrapped in a collagen membrane and functionalized with growth factors, pharmacological agents or axon growth promoting cells into complete or partial SCI of the adult rat thoracic spinal cord [18, 19]. They reported moderate to substantial collagen deposition and cell migration into the collagen scaffolds by 2–6 weeks after implantation. Cell infiltration was suggested to be due to meningeal fibroblasts but this notion was made, largely, on the basis of histological stains and the authors conceded that other sources of fibroblast-like cells were possible since the dura mater had been compromised. The present work used immunohistochemistry to demonstrate substantial invasion of the implanted scaffolds by vimentin-positive, GFAP-negative and S100-negative process-bearing cells that were often orientated along the longitudinal axis of the scaffold, following the microporous structure of the scaffold. This phenotype is typical for fibroblast-like cells, however, an unequivocal identification of fibroblasts by specific or unique immunohistochemical markers remains problematic in tissue sections of complex lesions (e.g. [41, 42]). Still, the phenotype described here precludes the involvement of the two of the major CNS and PNS glia (i.e. astrocytes and SC) both of which express all three antigens to varying degrees [43, 44].

The migration of fibroadhesive cells into spinal lesions and their expression of ECM molecules, as well as axon growth-repulsive molecules (e.g. semaphorin III and CSPGs), results in a fibrotic lesion core that is non-permissive for axon regeneration [45–47]. Numerous sources have been described for the migrating fibroblasts, such as the meninges (e.g. [8, 45]) as well as perivascular regions from large and small blood vessels, including pericytes [42, 48]. It is possible that such reactive fibroblast-like cells may have used chemotactic cues to migrate along the micro-structured surface of implanted scaffolds. *In vitro* studies have demonstrated that collagen types I, II and III (and their degradation products) are chemotactic for fibroblasts [49].

The multiple layers or sheets of vimentin-positive, GFAP-negative, S100-negative fibroblasts located at the transition zone of the implant–host interface appeared to isolate the scaffold from the surrounding reactive host tissues, as evidenced by the minimal penetration of reactive astrocytic processes or axonal profiles. A clear anatomical interface has been reported to form *in vivo* and *in vitro* where reactive astrocytes encounter migrating fibroblasts [8, 9, 50]. *In vivo* studies have suggested that membrane bound signalling molecules such as astrocytic Ephrin-B2 and EphB2 receptors on fibroblast may play an integral cell-contact mediated role in the mutual territorial exclusion seen at the astroglial–meningeal fibroblast interface after SCI [8]. Similarly, Novikova *et al.* demonstrated reactive astrogliosis that was separated, to some extent, from an implanted, biodegradable polyhydroxybutyrate scaffold by a substantial region of DAPI-labelled, GFAP-negative cells [51]. More recently, scaffolds made of chitosan, alginate, or a combination of chitosan and alginate were implanted into experimental thoracic spinal cord injuries of the adult rat and GFAP-immunohistochemistry demonstrated a largely GFAP-negative area surrounding all implants, however, the authors did not perform a nuclear counterstain or double immunohistochemistry to demonstrate the extent or identify the type of cells occupying this region. They were, therefore, unable to comment on the potential relevance of the observation on implant–host integration (their Fig. 6 [52]). The similarities between the above examples of host tissue responses to scaffold implantation and those demonstrated in the present investigation are striking. This might suggest that the phenomenon reported in the present investigation extends beyond the use of collagen-based scaffolds in experimental SCI and highlights an encapsulating aspect of the host response that is detrimental to the intended bridging role of the implanted engineered substrate.

The development of fibrotic encapsulation around implanted devices or materials has been reported to depend on the type of polymer being used, but when present has been shown to interfere with device performance [53, 54]. Fibrotic encapsulation has been shown to interfere with the release of bioactive molecules from drug delivery systems implanted into CNS tissues [55, 56], or to progressively reduce electrical contact between implanted electrodes and host tissues (for review see [57]). In the context of nervous tissue repair, fibrotic encapsulation of silicon-based conduits (used for the repair of PNS injuries in experimental animals and even in clinical applications) has been reported to result in the chronic development of constriction of the regenerated nerve, leading to demyelination and even Wallerian degeneration of the regenerated nerve (e.g. [58]). In some instances, encapsulation of a bioengineered implanted into experimental spinal cord lesions has been shown to interfere with regenerating host axons, effectively re-routing the axons around rather than through the scaffold [59]. Although such scarring activity has a detrimental effect on the intended role of the implant, relatively little attention has been focussed on this aspect of the host’s cellular response to implanted bioengineered scaffolds in SCI.

As demonstrated here, the presence and extent of fibroblast-like encapsulation at the implant interface may have consequences on the quantification of other parameters of host scarring, such as astrogliosis. The extent of local astrogliosis has a major impact on the ability of sprouting/regenerating axons to interact with the scaffold, and numerous qualitative and quantitative investigations (including our own) have reported that the implantation of type-I collagen scaffolds [31, 33, 60] (or scaffolds derived from other natural or synthetic polymers [61, 62]), reduce astrocytic scarring around the implants. The present investigation demonstrates the influence of the choice of positioning of the AOI on the outcome of morphometric analysis: positioning the edge of the AOI at the edge of the implant results in a lower value for GFAP-positive astrogliosis being measured than that obtained by positioning the AOI at the clearly defined edge of the astrocytic scarring. This effect is due to the inclusion of the territory occupied by the GFAP-negative fibroblast-like transition zone into the quantification, and the subsequent skewing of the data to lower values. However, the strict comparison of only GFAP-containing territories indicated that there was no scaffold-induced reduction in the extent of astroglial scarring at the graft–host interface. The adoption of strict anatomical standards and a clear description of how the positions of the AOI are applied are critical for ensuring the reproducibility of data acquisition and for valid comparisons between the data sets reported by different research groups.

The lesion and implantation procedure initiates a stereotypical pattern of complex cellular and molecular responses to injury. This pattern includes blood vessel damage, bleeding and oedema, the deposition of a fibrin clot and the recruitment of sequential waves of neutrophils, monocytes and macrophages, primarily from haematogenous sources [46]. These inflammatory cells regulate the process of fibrosis by producing a variety of cytokines (including IL-1, bFGF, PDGF, TGF-β) which directly activate and recruit fibroblasts, and induce astroglial activation to secrete proteoglycans, glycoproteins and other molecules that help lay down the extracellular matrix at glial-fibroblast scarring interfaces (reviewed by [53, 54, 63, 64]).

Quantitative analysis of activated microglia around implanted collagen scaffolds revealed an overall reduction in comparison to the tissues surrounding the lesion-only control group. The apparent effect was primarily due to reduced IBA-I-immunoreactivity in the lesioned white matter tract areas rostral and caudal to the implant. In contrast to the reduction of IBA-I-immunoreactivity in the damaged host tissues around the implant, quantification of IBA-I-immunoreactivity within the implant revealed that substantial recruitment and migration of inflammatory cells had taken place into the scaffold. However, many of the IBA-I-immunoreactive cells had adopted morphological features (such as the extension of processes) that were more characteristic of resting microglia than of classic, rounded, phagocytic macrophages. The reason for the reduction of inflammatory cells around the implanted scaffold is unknown but it is possible that the implant may have physically stabilized the severed rostral and caudal ends of the lesioned white matter tracts, and that this in turn resulted in reduced tissue degeneration. The surprising, process bearing phenotype of the IBA-I-positive cells within the implanted scaffold is a novel observation and may reflect the influence of the scaffold polymer (type-I collagen) or the scaffold architecture on cell behaviour. In the PNS, type VI-collagen has been demonstrated to influence repair by supporting macrophage migration and polarization towards the M2 phenotype [65]. Scaffolds derived from other ECM sources have also been reported to induce a switch of macrophage polarization from M1 to M2 [66–68]. The chemical cross-linking of such scaffolds has not only been shown to reduce the rate of scaffold degradation but also to inhibit the M2 polarization and to induce scar formation [66]. The continued presence of the scaffold by 10 weeks after implantation, as indicated in the present study, suggests that the degradation of the scaffold was a particularly slow process, most likely caused by the extent of cross-linking that was employed [26]. Future studies will be required to determine the spatio-temporal polarization of recruited macrophages after the implantation of such bioengineered scaffolds and their relevance to tissue scarring or repair.

A detailed understanding of how implanted scaffolds interact with host tissues remains to be determined. However, recent multi-component intervention strategies, including the use of micro-structured collagen in combination with a range of neutralizing molecules, growth factors and local cAMP injections have given cause for some optimism, highlighting partial success in enhancing functional recovery, with reduced astroglial scarring and increased axon regrowth within the lesion/implantation site as possible mechanism of action (e.g. [69] and reviewed in [70]). The authors have also strongly suggested enhanced neurogenesis within the lesion/implantation site as a novel response to the combined intervention strategy but failed to unequivocally demonstrate new neuron formation using βIII-tubulin immunohistochemistry. Our own earlier study used immunohistochemistry for NF200 as well as for MAP2a/b to study the responses of host neuronal profiles to collagen scaffold implantation. No evidence of neurogenesis could be observed although some MAP2a/b-positive dendritic profiles could occasionally be observed penetrating the scaffold [31]. The combined strategy of Li *et al.* could only induce a poor degree of astroglial integration into the scaffolds. However, potential interference of implant–host integration by fibroblast-like cell encapsulation was not explored [69].

## Conclusions

The present observations strongly suggest that the host scarring response to the implantation of a microstructured type-I collagen scaffold includes a substantial fibroblast-like encapsulation component. The identification of the cellular and molecular mechanisms that influence implant–host integration, particularly those affecting fibroblast-like encapsulation at the implant interface, is pivotal for the future development and implementation of such scaffolds in bridging strategies for SCI. Future interventions that are capable of reducing encapsulation type responses are likely to facilitate greater implant–host integration and improve functional tissue repair, bringing closer the prospect of bioengineered scaffolds contributing to multi-functional strategies in tissue engineering and regenerative medicine following traumatic SCI.

## Ethical approval

Animal care and experimental procedures were carried out in accordance with the guidelines of the Belgian animal protection statute and were approved by the Belgium governmental ethical committee.
